# Enhanced Oral Bioavailability and Biodistribution of Voriconazole through Zein-Pectin-Hyaluronic Acid Nanoparticles

**DOI:** 10.1021/acsami.4c16326

**Published:** 2024-12-19

**Authors:** Margani Taise Fin, Camila Diedrich, Christiane Schineider Machado, Letícia Marina da Silva, Ana Paula Santos Tartari, Isabella Camargo Zittlau, Samila Horst Peczek, Rubiana Mara Mainardes

**Affiliations:** † Laboratory of Nanostructured Formulations, Universidade Estadual do Centro-Oeste-UNICENTRO, Alameda Élio Antônio Dalla Vecchia, 838, 85040-167 Guarapuava, PR, Brazil; ‡ Pharmacy Department, Universidade Estadual do Centro-Oeste-UNICENTRO, Alameda Élio Antônio Dalla Vecchia, 838, 85040-167 Guarapuava, PR, Brazil

**Keywords:** nanoparticles, zein, hyaluronic acid, pharmacokinetic, biodistribution, voriconazole

## Abstract

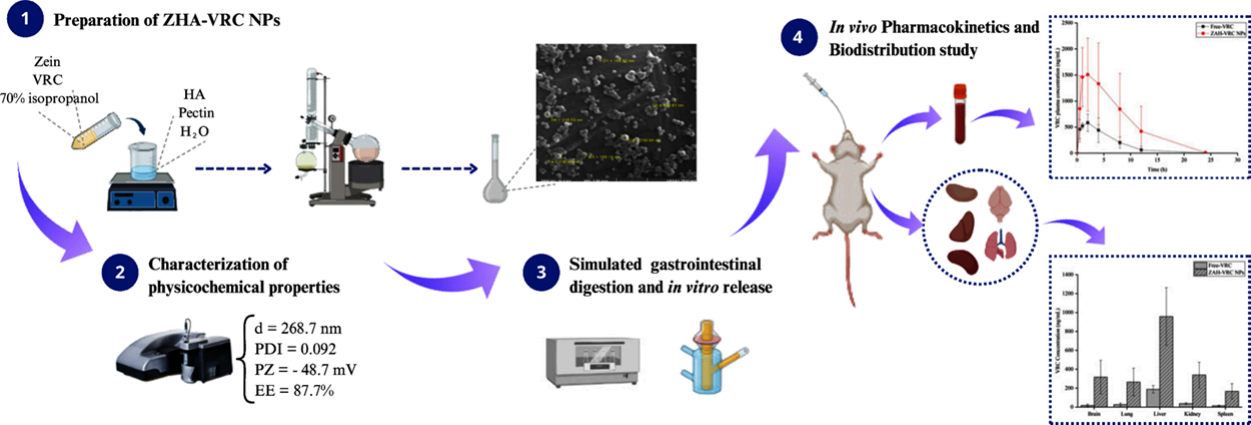

Nanotechnology-based drug delivery systems offer a solution to the pharmacokinetic limitations of voriconazole (VRC), including saturable metabolism and low oral bioavailability. This study developed zein/pectin/hyaluronic acid nanoparticles (ZPHA-VRC NPs) to improve VRC’s pharmacokinetics and biodistribution. The nanoparticles had a spherical morphology with an average diameter of 268 nm, a zeta potential of −48.7 mV, and an encapsulation efficiency of 88%. Stability studies confirmed resistance to pH variations and digestive enzymes in simulated gastric and intestinal fluids. The in vitro release profile showed a controlled release, with 8% of the VRC released in 2 h and 16% over 24 h. Pharmacokinetic studies in rats demonstrated a 2.8-fold increase in the maximum plasma concentration and a 3-fold improvement in bioavailability compared to free VRC. Biodistribution analysis revealed enhanced VRC accumulation in key organs. These results suggest that ZPHA-VRC NPs effectively improve VRC’s therapeutic potential for oral administration.

## 1 Introduction

Fungal infections are a major global public health concern, particularly affecting immunocompromised individuals, neonates, and patients recovering from SARS-CoV-2 infections.^1−4^ These infections are associated with high hospitalization rates, increased healthcare costs, and significant morbidity and mortality.^5−8^ Invasive fungal infections (IFIs), caused predominantly by *Cryptococcus sp.*, *Candida sp.*, and *Aspergillus sp.*, represent a critical challenge due to their severe clinical outcomes. Management of IFIs often relies on antifungal agents, with azole derivatives, such as voriconazole (VRC), playing a central role.^9,10^

Voriconazole (VRC) is a broad-spectrum azole antifungal derived from fluconazole, approval for clinical use in 2002. Its mechanism of action involves inhibiting ergosterol synthesis in the fungal cell membrane by disrupting cytochrome P450 enzyme activity,^9,11^ Despite its advantages, including high oral bioavailability and extensive tissue penetration, VRC faces substantial pharmacokinetic challenges. Its poor aqueous solubility (0.09 mg/mL), nonlinear pharmacokinetics due to saturable metabolism, and significant interindividual variability limit its clinical application.^12^ Additionally, existing formulations, such as oral tablets and intravenous solutions, are associated with adverse effects, including hepatotoxicity, nephrotoxicity, and low stability in physiological environments, further restricting its therapeutic potential.^9^

Nanotechnology has emerged as a promising strategy to address the limitations of conventional VRC delivery systems. Nanoparticle-based delivery platforms offer enhanced drug solubility, stability in gastrointestinal fluids,^13,14^ controlled release, improved mucosal adhesion, and deeper tissue penetration, thereby improving intestinal absorption,^14,15^ and bioavailability.^16^ Moreover, these systems can reduce systemic toxicity,^12,17^ providing a safer alternative for antifungal therapy.

Among nanotechnological approaches, polymeric, protein-based, and lipid-based nanoparticles have been explored to mitigate VRC’s low solubility and erratic pharmacokinetics. These technologies also expand administration possibilities, including topical, ocular, and pulmonary routes. However, despite these advancements, a significant gap remains in understanding the therapeutic efficacy of VRC-loaded nanoparticles in vivo, particularly concerning their pharmacokinetics and biodistribution.^9^ This limitation underscores the need for the development of new nanostructured systems for VRC delivery, which not only overcome existing pharmacokinetic challenges but also offer greater stability, improved tissue targeting, and reduced adverse effects.

Natural protein nanoparticles, such as albumin,^18^ zein^19^ and gliadin,^20^ stand out among nanostructured systems due to their biodegradability, biocompatibility, high binding capacity, effective cellular absorption, and amphiphilic characteristics.^21^ Zein-based nanoparticles stabilized with pectin and hyaluronic acid (HA) offer a novel solution to overcome VRC’s pharmacokinetic challenges. Zein, a natural protein derived from corn, has showed applications for drug delivery systems.^22^ However, limitations such as instability and aggregation under physiological conditions necessitate the use of stabilizing agents like pectin and HA to improve nanoparticle integrity and functionality.^23−26^ Pectin, a natural polysaccharide, enhances the stability, biocompatibility, and mucoadhesive properties of zein nanoparticles,^27^ while HA offers additional advantages, such as high affinity for mucin and cellular targeting through interactions with CD44 receptors.^28−31^ These properties make the combination of zein, pectin, and HA a promising strategy to overcome the challenges associated with VRC delivery.

This study aimed to develop and characterize zein-based nanoparticles stabilized by pectin and HA for VRC encapsulation. These nanoparticles were evaluated for their pharmacokinetic performance and biodistribution in rats following oral administration. By enhancing VRC bioavailability and addressing its pharmacokinetic limitations, this study demonstrates a significant advancement in antifungal drug delivery and provides a foundation for the clinical application of nanostructured systems in the treatment of IFIs.

## 2 Materials and Methods

### 2.1 Materials

Voriconazole (≥98% purity), Fluconazole (≥98% purity), Zein, Pectin from apple (50–75% esterification, 65% galacturonic acid content), Type II mucin, Formic acid, Pepsin from porcine gastric mucin, and Pancreatin from porcine pancreas were purchased from Sigma-Aldrich (St. Louis, MO, USA). Hyaluronic acid (50–90 kDa) was purchased from Contipro (Czech Republic). HPLC-grade acetonitrile (ACN) and methanol were purchased from Merck (São Paulo, Brazil). Isopropanol, Polysorbate 80, Sodium pyrophosphate, and Monobasic potassium phosphate were purchased from Synth (São Paulo, Brazil). Chloride acid and Acetic acid were purchased from Vetec (Duque de Caxias, Brazil). Ultrapure water was produced using a Millipore purification system (Burlington, MA, USA). Potassium chloride, Sodium chloride, Sodium hydroxide, Monobasic sodium phosphate, and Dibasic sodium phosphate were purchased from Biotec (São Paulo, Brazil). Ketamine and Xylazine were purchased from Ceva (Paulínea, Brazil).

### 2.2 Preparation of Zein-Pectin-Hyaluronic Acid Nanoparticles Containing Voriconazole

ZPHA-VRC NPs were prepared using a modified nanoprecipitation method with adaptations.^32,33^ Briefly, the organic phase consisted of zein and VRC, both dissolved in 70% isopropyl alcohol at pH 5.7. This solution was incubated in a shaker at 100 rpm and 25 °C for 2 h. The aqueous phase comprised hyaluronic acid and pectin at pH 4.0. The organic phase (2 mL) was gradually added to the aqueous phase (10 mL) under continuous stirring at 600 rpm for 30 min.

The organic solvent was removed by evaporation under vacuum at 55 °C and 100 rpm for 10 min. The final volume was adjusted to 10 mL with ultrapure water. An aliquot of 1 mL was ultracentrifuged at 15,000 rpm and 25 °C for 30 min, and the supernatant was stored under refrigeration (2–8 °C) for subsequent quantification of the free drug. The nanoparticles were stored under refrigeration (2–8 °C) and protected from light. The same procedure was performed for preparing drug-free nanoparticles (ZPHA NPs). The nanoparticles were lyophilized without a cryoprotectant agent for subsequent solid-state characterization analyses. The samples were stored in a desiccator.

### 2.3 Characterization of ZPHA-VRC NPs

The particle size and dispersity were determined using the dynamic light scattering technique (BIC 90 Plus, Brookhaven). The samples were diluted in ultrapure water and analyzed with a scattering angle of 90°, at 25 °C and a laser wavelength of 659 nm. Measurements were performed in triplicate, and data were expressed as mean value ± standard deviation.

The zeta potential analysis was performed using a ZetaSizer Nano ZS (Malvern) by determining the electrophoretic mobility. The nanoparticles were diluted in a 1 mM KCl solution to obtain a uniform dispersion with good scattering intensity. The result was obtained through the average of three repetitions and expressed as mean value ± standard deviation.

The morphological analysis of the nanoparticles was performed using scanning electron microscopy (SEM). The nanoparticles were diluted in ultrapure water at a ratio of 1:100, deposited on a metal support with carbon tape and kept at room temperature for 24 h. After drying, the sample was metalized with gold, and the samples were stored in a desiccator until the analyses were performed. Images were obtained using a SEM (VEGA3 instrument from Tescan), operating at 30 Kv and up to 40.000 factor magnification.

FTIR spectra of samples were analyzed by scanning in the wavelength region between 500 and 4000 cm^–1^ using an infrared spectrophotometer in ATR mode (Frontier, PerkinElmer).

An X-ray diffractometer (D2 Phaser, Bruker) was used to establish the diffraction pattern of samples. The measurement parameter included the angle range of 2θ, with a sweep from 5 to 60°, using a copper Kα radiation tube (λ = 1.5418 Å), a current of 10 mA, and a voltage of 30 kV. The samples were previously frozen in a freezer −20 °C for 24 h, and submitted to liophilization for 24 h.

### 2.4 Entrapment Efficiency

Entrapment efficiency (EE) was determined through an indirect method, following the quantification of VRC by HPLC (Alliance 2695, Waters) with photodiode array detector. An aliquot of the supernatant from nanoparticles ultracentrifugation was diluted in methanol, filtered through a 0.22 μm pore membrane, injected into the system, and analyzed at a wavelength of 255 nm for determination of free VRC. Chromatographic separation and detection were achieved using a C18 column (XTerraMS, 5 μm; 4.6 × 250 mm) at a column temperature of 25 °C, with a flow rate of 1 mL/min in isocratic mode. The mobile phase consisted of acetonitrile:water:formic acid in the ratio 60:40:0.5 (v/v/v). The injection volume was 15 μL, with retention time of 3.8 min, and the total run time was 5 min.

The EE was determined according to eq 1, considering the initial drug concentration and the free drug concentration present in the stored supernatant.
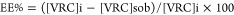
1where, [VRC]i corresponds to the initial concentration of voriconazole added to the formulation, and [VRC]sob represents the concentration of voriconazole present in the supernatant determined by HPLC. Experiments were performed in triplicate and data were calculated as mean value ± standard deviation.

### 2.5 Simulated Gastrointestinal Digestion

ZPHA-VRC NPs (containing 1.58 mg/mL of VRC) were dispersed in 1 mL of simulated gastric fluid (SGF; 50 mM KCl, 1% pepsin, pH = 1.2) and incubated at 37 °C under agitation at 150 rpm for 120 min. After 30-, 60-, and 120 min samples were centrifuged (15,500 rpm, 25 °C, 15 min), the supernatant was collected for analysis and the precipitate was resuspended again in a new freshly SGF. After, 2 h samples were suspended in simulated intestinal fluid medium (SIF; 50 mM KH_2_PO_4_, 15 mM NaOH, 1% pancreatin, pH = 6.8) and incubated at 37 °C under agitation at 150 rpm during 240 min. The samples were collected at 1, 2, 3, and 4 h for analysis. The supernatants were filtered through a 0.45 μm filter, diluted if necessary, and VRC released over time was quantified by HPLC.

### 2.6 In Vitro Release Study

The VRC release profile from ZPHA-VRC NPs was conducted using a Franz cell (7.0 mL receiver compartment and 1.5 cm diameter diffusion area) in 50 mM saline phosphate buffer (PBS) (pH = 7.4) containing 1% (m/v) polysorbate 80, maintaining at 37 °C with a stirring speed of 300 rpm (Santos et al., 2018). Polysorbate 80 was used to maintaining the sink conditions. Before analysis, the artificial nitrocellulose membrane (0.45 μm, Merck Millipore) was kept for 20 min in the receiver medium for equilibration and then fixed in the diffusion cell. The receiving chamber was filled with 7 mL of receiving medium. A 250 μL aliquot of nanoparticles (equivalent to 1050 μg of encapsulated VRC) was added to the donor chamber. At the pre-established times (0.5, 1, 2, 4, 8, 12, and 24 h), 1 mL of the receptor medium was withdrawn, and the same volume of medium was replaced. The samples were filtered through a 0.45 μm filter, and the cumulative amount of VRC release at different time points was evaluated by HPLC. In vitro, drug release data were fitted to different kinetic models to understand the mechanism and kinetics of VRC release.

### 2.7 In Vivo Pharmacokinetics and Biodistribution Study

#### 2.7.1 UPLC-MS/MS Conditions

Ultraperformance liquid chromatography (Acquity UPLC© Waters, Milford, MA, USA), coupled with a triple quadrupole mass spectrometer (XEVO-TQD, Waters) equipped with a Z spray electrospray ionization source (Waters, Milford, MA, USA), was employed to detect and quantify VRC levels in rat plasma and tissues after treatment. An ACQUITY UPLC BEH C18 reversed-phase chromatographic column (50 × 2.1 mm; 1.7 μm) was used, with a mobile phase composed of acetonitrile and water in a ratio of 70:30 (v/v), eluted at a flow rate of 0.2 mL/min in isocratic mode. The injection volume was 2 μL and the total run time was 3 min.

The samples were maintained at a temperature of 10 °C in the sampler, while the column oven was maintained at 40 °C. Detection by mass spectrometry was carried out in positive electrospray ionization (ESI+), in multiple reaction monitoring (MRM) mode, with a capillary voltage of 3.20 kV, gas flow in the 34 L/h, desolvation temperature of 350 °C, and desolvation flow rate of 400 L/h. The transitions set at *m*/*z* 350.02 → 126.98, and for internal standard (IS) fluconazole *m*/*z* 307.01 → 220.0 and 238.0.

The limits of quantification and detections were 1.23 and 4.12 ng/mL, respectively.

#### 2.7.2 Animals

Twenty-four male Wistar rats (250–300 g) were used in the study. The animals were housed in plastic cages with free access to food and water, maintained on a 12 h light/dark cycle, and kept at a constant temperature of 22 ± 1 °C. The study was conducted following approval by the Animal Use Ethics Committee of the Universidade Estadual do Centro-Oeste – Brazil (Approval number 047/2021) and the National Research Council’s Guide for the Care and Use of Laboratory Animals. Before the study, the animals were fasted overnight for 12 h and continued fasting for up to 4 h after dosing the formulations.

#### 2.7.3 Pharmacokinetics and Biodistribution: Treatment and Analysis

The pharmacokinetic study was divided into 2 groups (*n* = 6). The animals in groups 1 (ZPHA-VRC NPs in aqueous suspension) and 2 (Free-VRC dispersed in 0.9% NaCl) received a single dose of 15 mg/kg VRC by oral gavage. For the biodistribution study, the same dose was administered by oral gavage, performed with 2 groups (*n* = 3), group 1B received ZPHA-VRC NPs in suspension, and group 2B received free-VRC dispersed in 0.9% NaCl.

Blood samples were collected at predetermined time intervals (0.25, 0.5, 1, 2, 4, 8, 12, and 24 h) for the pharmacokinetic study. In the biodistribution study, a single blood collection was performed at 4 h, subsequently, the animals were sacrificed, and the organs (brain, lung, liver, kidney, and spleen) were collected. The animals were anesthetized with Ketamine (75 mg/kg) and Xylazine (10 mg/kg) and euthanized. The collected blood was centrifuged at 3500 rpm, 25 °C for 15 min to separate the plasma. The plasma samples were kept at −80 °C until analysis.

The collected tissues were homogenized with phosphate-buffered saline (PBS) pH 7.4 using a Turrax homogenizer (Marconi) at 33,000 rpm in three cycles of 30 s each. Protein separation (plasma or tissue homogenate) was performed by adding 500 μL of sample to 1000 μL of acetonitrile, vortexing (Chang Bioscience), followed by centrifugation at 14,000 rpm and 4 °C for 10 min. Samples were filtered through 0.22 μm hydrophobic PVDF filters (Millipore) and analyzed by UPLC-MS/MS. The calculated pharmacokinetic parameters included peak plasma concentration (*C*_max_), time of peak plasma concentration (*T*_max_), area under the VRC plasma concentration versus time curve (AUC_0–24h_), area under the concentration curve plasma concentration of VRC versus time extrapolated to infinity (AUC_0–∞_), relationship between areas (area), elimination half-life (*T*_1/2_), elimination constant (*K*_el_), apparent volume of distribution (Vd) and clearance (Cl).

### 2.8 Statistical Analysis

All studies were performed in triplicate (except pharmacokinetics) and data were expressed as mean and standard deviation (mean ± SD). One-way analysis of variance (One-way ANOVA) was used, followed by Tukey’s post-test with a 95% confidence interval. Results were considered statistically different if ρ < 0.05. The software used for statistical analysis and graphing included MINITAB, ORIGINPRO, and STATISTICA (Minitab, 2021; OriginPro, 2018; Statistica, 2007).

## 3 Results and Discussion

### 3.1 Preparation and Characterization ZPHA-VRC NPs

#### 3.1.1 Size, Dispersity, Zeta Potential, EE, and Morphology

Nanoparticles were prepared using the nanoprecipitation method, a well-established technique for obtaining protein nanoparticles.^33,34^ The method involved precipitation the zein from an ethanolic phase into an aqueous phase containing the stabilizing agent pectin, leading to the formation of nanoparticles. HA was included in the aqueous phase to structure the formulation and enhance intestinal absorption of the nanoparticles via CD44 receptors. Nanoprecipitation is favored for its simplicity, scalability, and ability to produce nanoparticles with controlled size and morphology. The zein, pectin and hyaluronic acid nanoparticles were successfully synthesized using this method, ensuring uniformity and reproducibility in the particle characteristics.

Initially, the amounts of zein and HA were varied across three levels to optimize particle size. The smallest particle size (191 ± 6 nm) was achieved when the two components were used in equal proportions. Subsequently, the amount of pectin was varied at two levels, with both levels contributing to an increase in particle size (313 ± 8 nm). Despite this increase, pectin’s stabilizing role makes its presence crucial in the formulation. Moreover, a modification in the solvent evaporation method significantly influenced particle size. When the evaporation technique was shifted from SpeedVac to rotary evaporation the particle size was notably reduced (273 ± 8 nm). This adjustment underscores the importance of processing parameters in achieving optimal nanoparticle characteristics.

The characterization data presented in Table 1 indicate that the mean particle diameters range from 250 to 290 nm. Unloaded nanoparticles had a similar size to the ZPHA-VRC NPs (*p* > 0.05). These values are comparable to those obtained in the study by Chen et al., which evaluated the production of zein nanoparticles and the influence of adding HA or its salt with different molecular weights.^35^

**Table 1:** Mean Diameter, Dispersity, Zeta Potential, and EE of Zein-Pectin-Hyaluronic Acid Nanoparticles Containing Voriconazole (ZPHA-VRC NPs) and Unloaded Zein-Pectin-Hyaluronic Acid Nanoparticles (ZPHA NPs) (*n* = 3)

parameter	ZPHA-VRC NPs	ZPHA NPs
mean diameter (nm)	268.7 ± 12.5^b^	282.6 ± 6.1^b^
dispersity	0.092 ± 0.036^b^	0.136 ± 0.022^b^
zeta potential (mV)	–48.7 ± 0.9^b^	–47.4 ± 3.0^b^
EE (%)^a^	87.7 ± 1.9	

a EE = Entrapment efficiency.

b Same letters mean statistical equality and different letters statistical difference (ANOVA and post-Tukey test and ρ < 0.05). They were analyzed by column.

**Figure 1:**
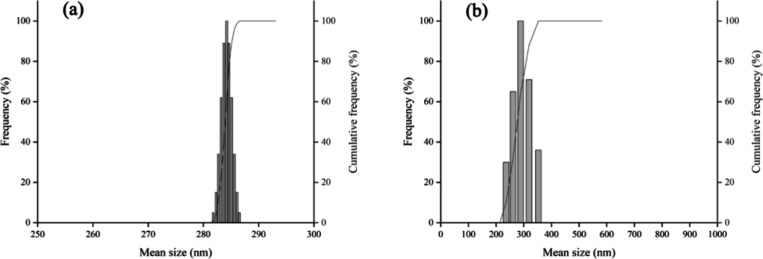
Particle size distribution of ZPHA NPs (a) and ZPHA-VRC NPs (b).

**Figure 2:**
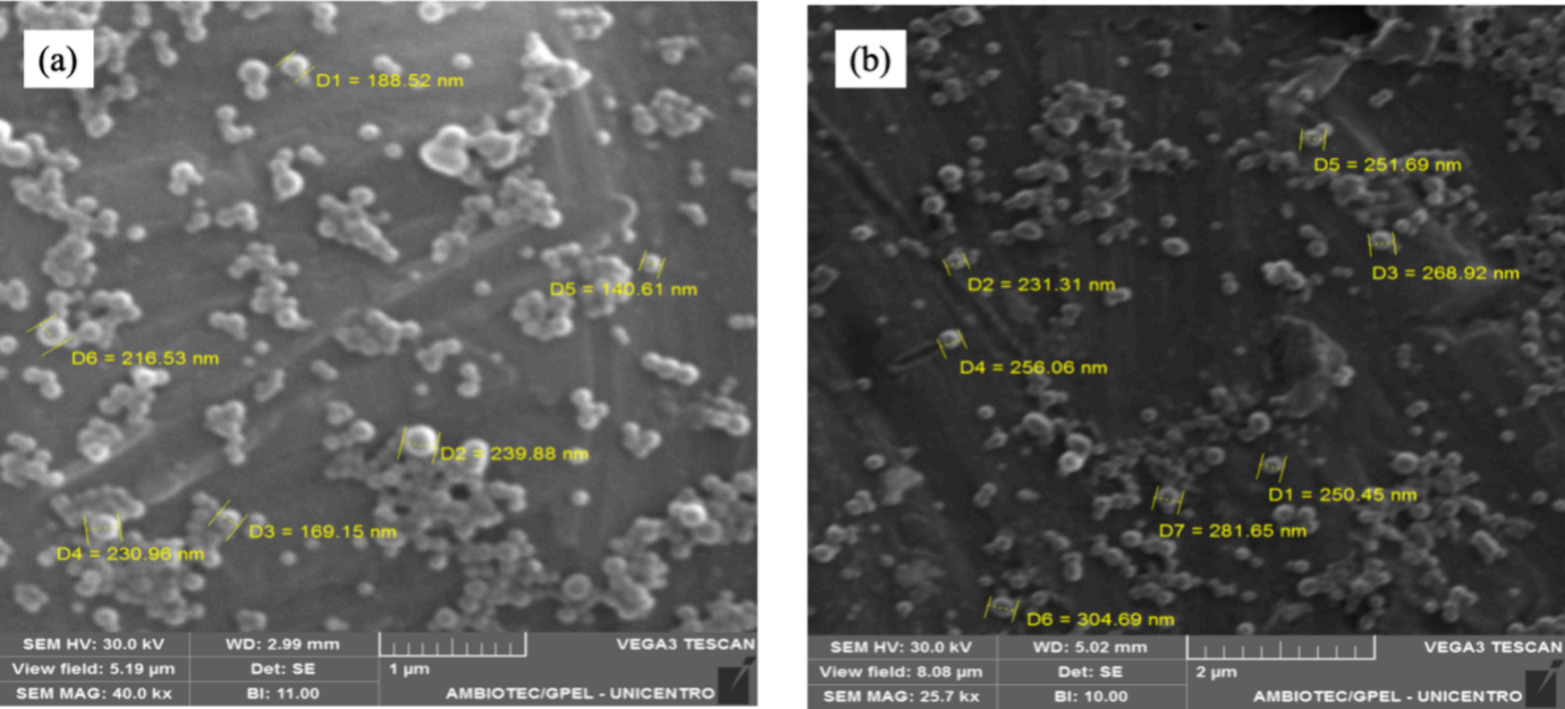
SEM images of: (a) ZPHA NPs; and (b) ZPHA-VRC NPs.

Both unloaded (Figure 1a) and loaded nanoparticle formulations (Figure 1b) exhibited a monomodal size distribution profile, as confirmed by dynamic light scattering (DLS) (Figure 1). The lower dispersity values and high negative zeta potential indicate a narrow size distribution and good stability of the formulations. SEM images revealed that ZPHA NPs (Figure 2a) and ZPHA-VRC NPs (Figure 2b) were spherical and with homogeneous size distribution.

The entrapment efficiency (EE) for was 87.7%, highlighting the high encapsulation capacity of zein nanoparticles. This high EE aligns with other studies demonstrating zein’s ability to encapsulate significant amounts of drug due to its amphiphilic nature.^36−42^

The physicochemical properties of nanoparticles, including particle size, dispersity, and zeta potential, play a crucial role in determining their absorption efficiency. Drug absorption in the gastrointestinal tract can occur through several mechanisms: passive diffusion across cell membranes, where smaller nanoparticles can pass through the lipid bilayer; paracellular transport through tight junctions, which allows for the passage of slightly larger particles; and transporter-mediated uptake, where nanoparticles interact with specific transport proteins on the cell surface. Nanoparticles within the size range of 20–1800 nm are particularly favorable for these absorption pathways, with smaller nanoparticles often being absorbed more efficiently via passive diffusion, while larger nanoparticles may utilize endocytosis or paracellular transport for absorption.^43^

The negatively charged surface is particularly significant in the gastrointestinal tract (GIT), where it can interact with mucin, the primary glycoprotein component of mucus. Mucin itself is also negatively charged, which might initially suggest that electrostatic repulsion would occur between HA-coated nanoparticles and the mucosal layer. However, the interaction between HA and mucin is more complex. HA can interact with mucin via hydrogen bonding and hydrophobic interactions, rather than relying solely on electrostatic attraction. These interactions facilitate the adhesion of HA-coated nanoparticles to the mucosal surface, enhancing the retention time of the nanoparticles in the GIT and potentially improving the absorption of the encapsulated drug.^44^ Moreover, the mucoadhesive properties of HA can be advantageous for drug delivery as they can prolong the residence time of the nanoparticles in the absorption sites, allowing for sustained and targeted release of therapeutic agents. This interaction with mucin, despite the like charges, highlights the unique role of HA in nanoparticle-based drug delivery systems within the gastrointestinal environment.^28,45^

#### 3.1.2 Fourier Transform Infrared Spectroscopy (FTIR)

**Figure 3:**
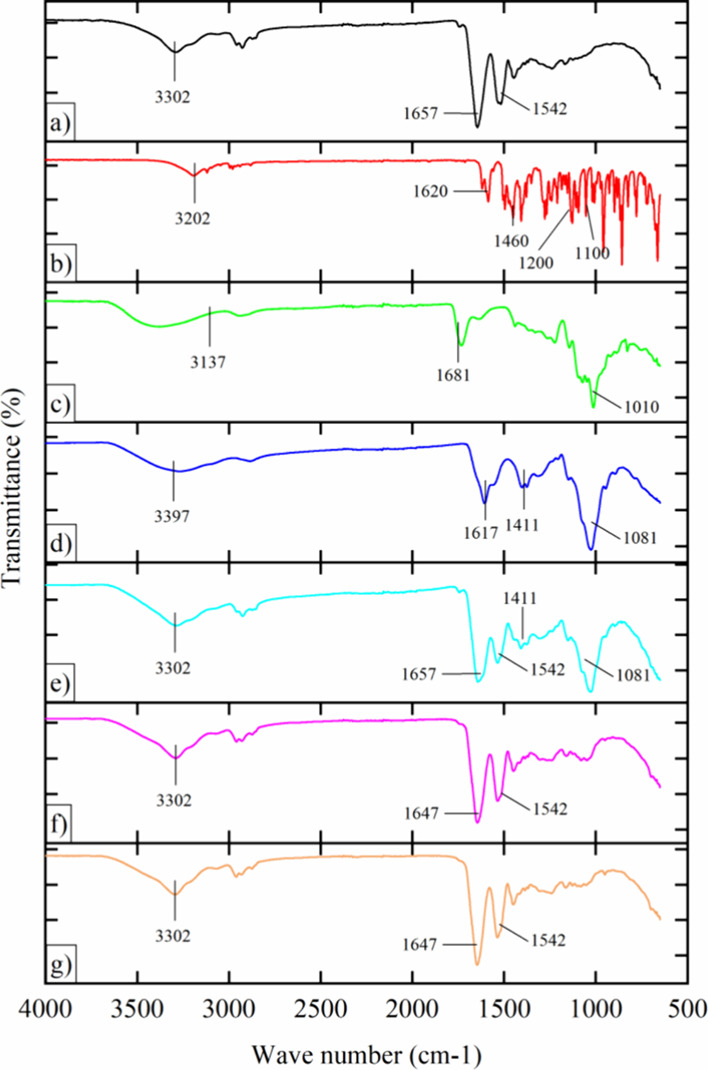
FTIR spectrum of: (a) Zein; (b) Voriconazole; (c) Pectin; (d) Hyaluronic acid; (e) Physical mixture; (f) ZHA NPs; (g) ZPHA-VRC NPs.

The findings derived from the FTIR spectroscopic analysis of the formulation components, physical mixture, and subsequent nanoparticle synthesis are presented in Figure 3. Within the zein spectrum (Figure 3a), distinctive bands were observed with vibrations centered at 3302 cm^–1^, indicative of hydroxyl group (−OH) stretching. The amide I (−C=O) and amide II (−C=N) functional groups were discerned at 1647 and 1542 cm^–1^, respectively.^37^ In Figure 3b, the VRC profile displayed vibrations at 3202 cm^–1^ (−OH), accompanied by peaks at 1620 and 1460 cm^–1^ corresponding to aromatic stretching (−C-C) and (C=N) bonds, respectively.^46^ Notably, characteristic bands associated with C–N and C–F stretching vibrations at 1200 and 1100 cm^–1^, respectively, were absent in ZPHA NPs and ZPHA-VRC NPs (Figure 3g). The pectin spectrum (Figure 3c) exhibited stretching vibrations at 3137 cm^–1^ (−OH), 1681 cm^–1^ (−C=O), and 1010 cm^–1^ attributed to stretching vibrations (C–O) derived from species containing C–O–C and C–H groups.^47^ HA (Figure 3d) demonstrated peaks at 3397 and 1617 cm^–1^, corresponding to hydroxyl groups (−OH) and amide I (−C=O), respectively, consistent with findings from other authors.^35,36,48^ The examination of the physical mixture (Figure 3e) facilitated the elucidation of compound interactions postnanoparticle formation.

The spectra obtained from ZPHA NPs (Figure 3f) and ZPHA-VRC NPs (Figure 3g) illustrate similar stretching vibrations observed at 3302, 1657, and 1542 cm^–1^, which correspond to hydroxyl, amide I, and amide II groups, respectively. The absence of vibrations at 1460 cm^–1^ indicates encapsulation of VRC within the nanoparticles. Notably, the shift in zein vibrations from 1657 to 1647 cm^–1^ (observed in Figure 3a,g) suggests the occurrence of hydrophobic interaction between zein molecules and VRC.^49^

#### 3.1.3 X-ray Diffraction (XRD)

XRD patterns for the samples reveal distinct characteristics that provide valuable insights into the crystalline and amorphous nature of the components and their interactions within the nanoparticle formulations.

**Figure 4:**
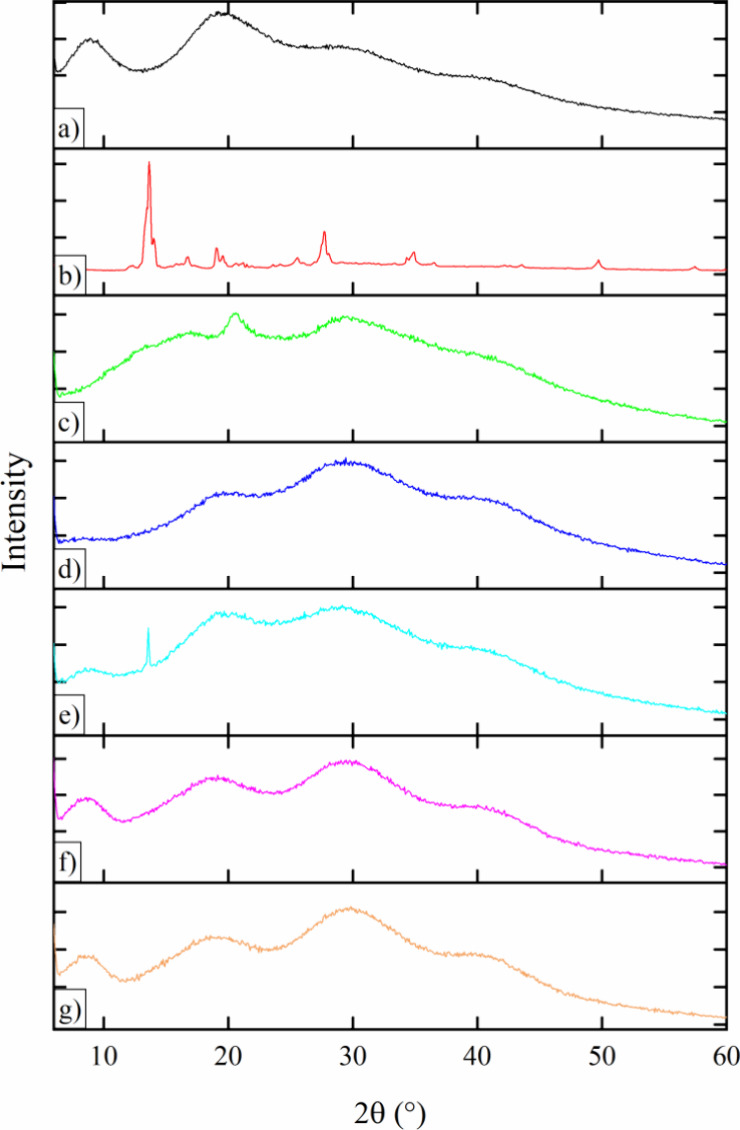
X-ray Diffractograms of: (a) Zein; (b) Voriconazole; (c) Pectin; (d) Hyaluronic acid; (e) Physical mixture; (f) ZPHA NPs; (g) ZPHA-VRC NPs.

The XRD pattern of zein (Figure 4a), being a proteinaceous substance, exhibited an amorphous material structure, evidenced by the presence of two broad peaks at diffraction angles (2θ) approximately 9° and 19°. Similarly, the XRD pattern of pectin (Figure 4c) displayed a comparable profile, characterized by broad peaks around 20° and 30°, indicative of its amorphous nature.^27^ HA (Figure 4d) also exhibits broad peaks around 2θ = 16.7° and 21.5°, reflecting its amorphous character. Contrarily, the VRC diffractogram (Figure 4b) revealed distinct and well-defined peaks at 2θ = 13.6°, 15.4°, 18.2° and 27°, which are characteristic of its crystalline structure.^50^ In the physical mixture (Figure 4e), the diffractogram displays a combination of sharp and broad peaks. The sharp peak of VRC (e.g., 2θ = 13.6°) are still visible, alongside the broader peaks from zein, pectin, and HA. This indicates that VRC retains its crystalline form in the physical mixture.

ZPHA-VRC NPs (Figure 4g) and ZPHA NPs (Figure 4f) exhibited a comparable amorphous profile. However, ZPHA-VRC NPs (Figure 4g) lacks the distinctive peaks associated with VRC crystallinity. This observation suggests the potential formation of a drug–protein complex, wherein VRC is dispersed within this system, likely resulting from amorphization induced by the nanostructure. Furthermore, there was a reduction in the intensity of the zein peak by 20°, potentially attributed to the presence of VRC and HA. The benefit of employing amorphous substances lies in their capacity to enhance solubility and absorption when in this state.^13,41^

### 3.2 Simulated Gastrointestinal Digestion

**Figure 5:**
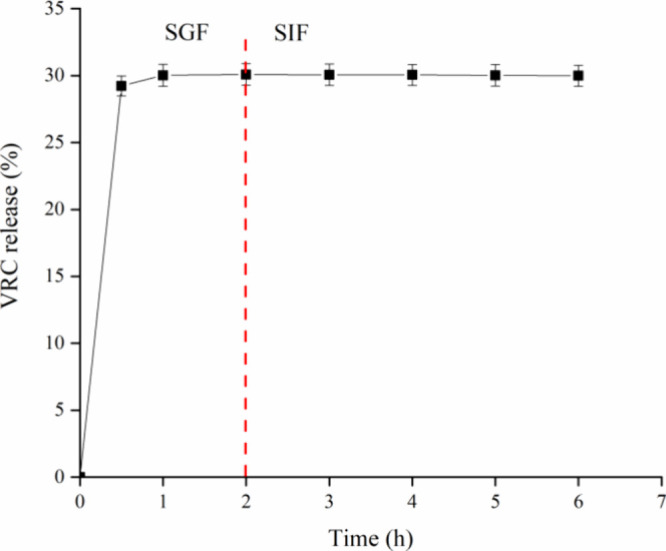
Release profile of VRC from ZPHA-VRC NPs in SGF (pH = 1.2) and SIF (pH = 6.8). The assay was conducted for 2 h in SGF, and an additional 4 h in SIF, at 37 °C and 150 rpm (*n* = 3).

The study on the release of VRC from nanoparticles in simulated gastrointestinal fluids aimed to predict their behavior postoral administration and their transit profile through the GIT. The results are shown in Figure 5.

The release profile of VRC in SGF indicated an initial burst release of approximately 28% within the first 30 min. This was followed by a slight increase to 30% within the first hour, which then stabilized and remained constant for up to 2 h. The initial rapid release could be attributed to the surface-bound drug that is readily dissolved upon exposure to the acidic environment. The constant release rate suggests a controlled release mechanism likely due to the interaction between VRC and the nanoparticle matrix, ensuring stability in the gastric environment. Upon transitioning to SIF (pH 6.8), VRC was not more released.

The nanoparticles managed to preserve 70% of the VRC payload. This high preservation rate suggests that ZPHA-VRC NPs efficiently safeguard VRC, facilitating its interaction with CD44 receptors in the intestine. This interaction likely enhances endocytosis and subsequent release of VRC into the tissues, underscoring the potential of these nanoparticles in improving oral drug delivery. Moreover, this protective mechanism is important for overcoming the challenges posed by the GIT, such as varying pH levels, enzymatic activity, and the presence of mucosal and epithelial barriers.^51^ Ensuring that a substantial amount of VRC remains intact until it reaches the target site enhances the therapeutic efficacy and reduces the frequency of dosing, thereby improving patient compliance and treatment outcomes.

### 3.3 In Vitro Release Study

**Figure 6:**
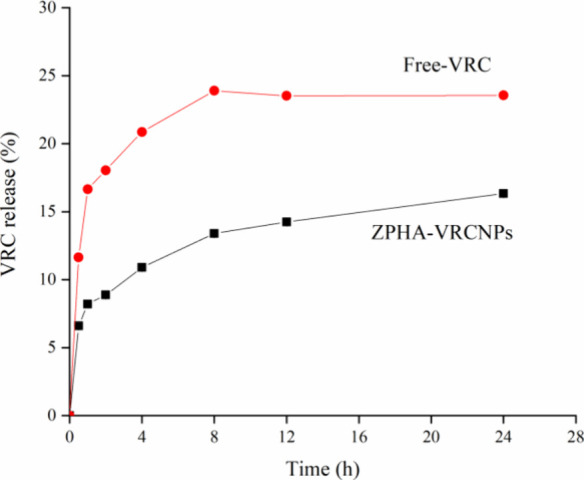
VRC release profile from ZPHA-VRC NPs and Free-VRC in PBS buffer solution (50 mM, pH 7.4, containing 1% polysorbate 80). The assay was conducted in a Franz cell at 37 °C and 300 rpm for 24 h (*n* = 3).

The VRC release profile from ZPHA-VRC NPs in PBS buffer (pH 7.4) is illustrated in Figure 6. To maintain sink conditions, 15% of the maximum solubility of VRC in the Franz cell was used, corresponding to 0.156 mg of VRC per mL of the receiving medium. For ZPHA-VRC NPs, an initial burst release of approximately 8% occurred within the first 2 h, followed by a sustained release phase that gradually increased to 16% after 24 h. This initial burst can be attributed to the desorption of VRC from the surface of the nanoparticles, a common phenomenon observed in nanoparticulate systems. The subsequent sustained release indicates the slow diffusion of VRC from the nanoparticle matrix into the surrounding medium. In contrast, free VRC demonstrated a significantly faster release, achieving approximately 25% within the same time frame, reflecting its unrestricted solubility and diffusion in the PBS buffer.

The slower and more controlled release observed with ZPHA-VRC NPs indicates effective encapsulation and gradual release, potentially enhancing the bioavailability and therapeutic efficacy of VRC. Such a controlled release system could help maintain therapeutic drug concentrations over prolonged periods, reducing dosing frequency and minimizing potential side effects associated with peak plasma concentrations. In summary, the data underscore the advantages of the nanoparticle formulation in modulating drug release, providing a sustained and controlled delivery profile, which is crucial for improving the pharmacokinetic and pharmacodynamic profiles of VRC.

Table 2 represents the data set acquired after the examination of the release profile via the utilization of the KinectDS software. This analysis relied on correlation coefficient values (r) to determine the kinetic model that elucidates the release mechanism of VRC facilitated by nanoparticles. The selection of the mathematical model was based on identifying the maximal *r* value among the employed models.

**Table 2:** Kinetic Models for the Release of VRC from ZPHA-VRC NPs in Saline PBS Buffer (50 mM, pH = 7.4, Containing 1% Polysorbate 80)

Kinect model	*r-value*
zero order	0.6378
first order	0.1182
second order	0.0999
Higuchi	–1.9977
Korsmeyer-Peppas	0.9868
Weibull	0.9873
Baker-Lonsdal	0.8777
Michaelis-Menten	1.0000
Hill’s equation	0.9877

Based on the data provided in Table 2, the release of VRC from nanoparticles is most accurately described by the Michaelis-Menten model, which demonstrated a perfect correlation (*r* = 1.0000). The other models also showed strong linearity, with correlation coefficients ranked as follows: Hill (*r* = 0.9877), Weibull (*r* = 0.9873), and Korsmeyer-Peppas (*r* = 0.9868). The release exponent value (*n* = 1.02) from the Korsmeyer-Peppas model suggests a Super Case II transport mechanism, as indicated by *n* > 0.89.

The high correlation coefficients observed in the Michaelis–Menten and Hill models imply that VRC binding occurs at specific sites on the adsorbent, particularly zein. This suggests first-order kinetics within the Michaelis–Menten framework and the possibility of more complex, higher-order interactions as described by the Hill model.^52,53^

Analyzing these *r* values reveals that each model provides insights into the release mechanism, encompassing diffusion, polymer relaxation (Korsmeyer-Peppas), and swelling (Weibull), consistent with non-Fickian transport dynamics (*n* > 0.89).^18,54,55^ This detailed evaluation highlights the critical role of understanding the complex dynamics of drug release from nanoparticulate systems, which is crucial for optimizing therapeutic outcomes and achieving desired pharmacokinetic profiles.

### 3.4 In Vivo Pharmacokinetics and Biodistribution Study

#### 3.4.1 Pharmacokinetics Analysis

**Figure 7:**
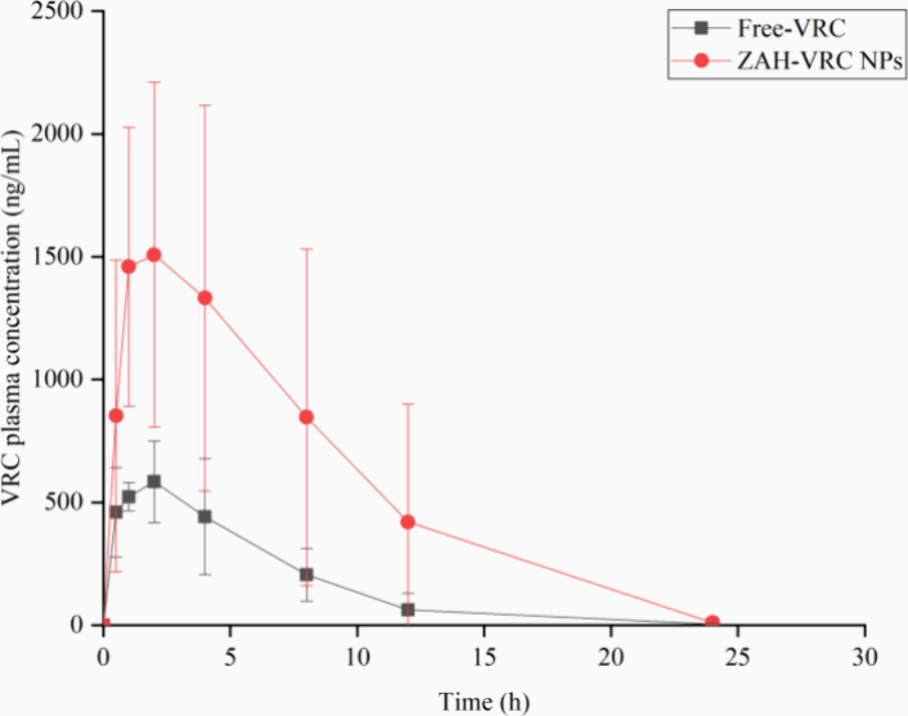
Plasma concentration–time curve following oral administration of 15 mg/kg VRC in male Wistar rats: Comparison of ZPHA-VRC NPs and free-VRC (*n* = 6).

The in vivo pharmacokinetic study was performed on male Wistar rats following the administration of VRC, either in its free form or encapsulated within nanoparticles, via a single oral dose. The primary objective was to evaluate the ability of the nanoparticles to modify the pharmacokinetic profile of VRC, thereby improving its bioavailability and distribution to key target organs. Figure 7 illustrates the plasma concentration of VRC over time following the administration of a single dose of either free VRC or ZPHA-VRC NPs, with the corresponding pharmacokinetic parameters summarized in Table 3.

**Table 3:** Pharmacokinetic Parameters after Oral Administration of a Single Dose (15 mg/kg) of ZPHA-VRC NPs or Free-VRC in Male Wistar Rats

parameter	free VRC	ZPHA-VRC NPs
*T*_1/2_ (h)	1.34 ± 0.39^a^	1.38 ± 0.73^a^
*C*_max_ (ng/mL)	657.1 ± 143.0^a^	1743.4 ± 701.5^b^
*T*_max_ (h)	2.2 ± 1.2^a^	2.8 ± 2.7^a^
*K*_el_ (L/min)	0.555 ± 0.178^a^	0.612 ± 0.283^a^
AUC_0–24_ (ng/mL/h)	3824.5 ± 1198.3^a^	11678.8 ± 6126.6^b^
AUC_0–∞_ (ng/mL/h)	3834.8 ± 1039.6^a^	11683.8 ± 5596.6^b^
*V*_d_ (L)	0.0056 ± 0.0031^a^	0.0025 ± 0.0024^a^
Cl (L/h)	0.0028 ± 0.0010^a^	0.0010 ± 0.0005^a^

a The results were expressed as mean ± standard deviation (*n* = 6).

b One-Way ANOVA, followed by Tukey's test, *p* < 0.05.

Upon analyzing the pharmacokinetic profile of VRC (Figure 7) through the plasma concentration curve, both formulations followed a similar trajectory. However, ZPHA-VRC NPs exhibited a significantly higher VRC concentration compared to the free drug. The peak plasma concentration (*C*_max_) was reached approximately 2 h after oral administration, followed by a gradual decline, with levels approaching zero within 24 h.

For a comprehensive understanding of the drug-organism interaction, additional pharmacokinetic parameters were analyzed, as delineated in Table 3. Upon comparing the pharmacokinetic parameters between ZPHA-VRC NPs and Free-VRC, it was determined that there were no significant differences in *T*_1/2_, *T*_max_, *K*_el_, *V*_d_, and Cl (*p* > 0.05). However, significant differences (*p* < 0.05) were observed in *C*_max_, AUC_0–24_, and AUC_0–∞_ between the two formulations.

The *C*_max_ of ZPHA-VRC NPs exhibited a 2.6-fold increase compared to Free-VRC, with respective attainment times of 2.8 and 2.2 h. This notable disparity in *C*_max_ was likewise evident in the results of AUC_0–*t*_ and AUC_0–∞_, which were 3-fold higher for the nanoparticles compared to the free drug, indicative of enhanced bioavailability of VRC when administered via ZPHA-VRC NPs.

The possible enhanced internalization of ZPHA-VRC NPs facilitated by CD44 receptors located in the intestinal mucosa, or the interaction between HA and mucin, contributes to elucidating the 3-fold increase in *C*_max_ and AUC for ZPHA-VRC NPs compared to Free-VRC.^56^ Similar findings have been reported in other studies where hyaluronic acid was used to enhance drug bioavailability.^50,57^ The most plausible mechanism underlying the mucoadhesive properties of HA is its capacity to engage in interactions and form hydrogen bonds with mucin.^28^

Enhancing the pharmacokinetics of VRC holds significant importance, given its narrow therapeutic index and considerable pharmacokinetic variability among individuals, resulting in nonlinear pharmacokinetics.^58^

#### 3.4.2 Biodistribution

**Figure 8:**
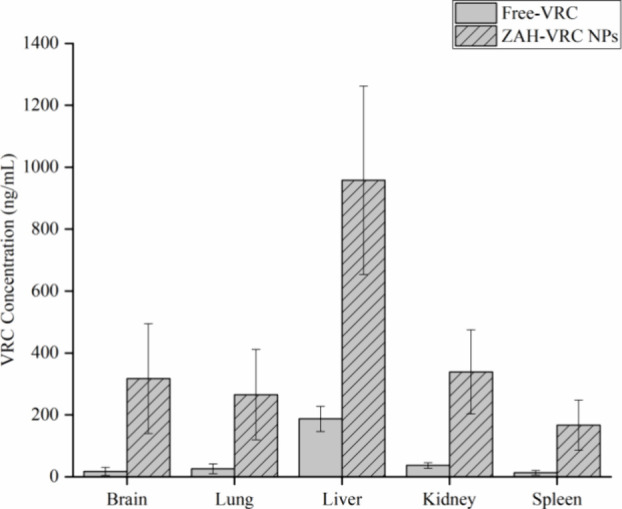
VRC concentration in brain, lung, liver, kidney, and spleen following a single oral dose (15 mg/kg) of ZPHA-VRC NPs or free VRC after 4 h of treatment (*n* = 3).

The biodistribution of VRC in male Wistar rats was analyzed to assess the drug’s distribution across various organs following oral administration of either Free-VRC or ZPHA-VRC NPs. Figure 8 presents the concentration of VRC in the brain, lungs, liver, kidneys, spleen, and heart 4 h after administration.

The results indicate that ZPHA-VRC NPs achieved significantly higher accumulation in key organs compared to Free-VRC. The liver exhibited the highest concentration of VRC, followed by the brain, lungs, and spleen. This pattern suggests that the nanoencapsulation of VRC enhances both its bioavailability and its ability to target specific tissues.

These findings are particularly relevant for the treatment of fungal infections such as systemic candidiasis, invasive pulmonary aspergillosis, and cryptococcal meningitis. The increased concentrations of VRC in the plasma, lungs, and brain imply that the nanoencapsulated formulation may offer superior therapeutic efficacy in these conditions.

The liver’s predominant VRC concentration aligns with its central role in drug metabolism via cytochrome P450 enzymes, although this also highlights the need for careful monitoring to mitigate the risk of hepatotoxicity.

The observed distribution pattern in the brain, lung, and liver is consistent with findings from previous studies. For instance, Smruthi et al., reported similar organ distribution when investigating naringenin encapsulated in PLA/PVA and zein/pectin nanoparticles administered orally, as opposed to its free form.^59^ Additionally, other author reported the enhanced accumulation in the liver, lungs, and kidneys when utilizing zein nanoparticles with and without HA particularly noting significant differences in tumor tissues due to the overexpression of CD44 receptors which facilitate the cellular internalization of HA.^33^

These findings underscore the potential of nanostructured systems to significantly enhance the biodistribution of VRC. The improved distribution across critical organs, such as the brain and lungs, could lead to better management of severe fungal infections, while the heightened liver concentrations necessitate cautious therapeutic monitoring to avoid adverse effects. Collectively, these studies support the potential of nanoparticle-based formulations to enhance the bioavailability and biodistribution of VRC, offering a pathway to more effective and safer antifungal treatments.

## 4 Conclusions

In this study, zein-pectin-hyaluronic acid composite nanoparticles containing voriconazole (ZPHA-VRC NPs) were successfully synthesized using the nanoprecipitation method for oral administration. The ZPHA-VRC NPs displayed favorable characteristics, including small particle size, uniform distribution, negative zeta potential, and a spherical shape. Voriconazole was effectively encapsulated in an amorphous state within the nanoparticles, which showed stability under simulated gastrointestinal conditions, ensuring controlled release. The in vivo pharmacokinetics and biodistribution studies in rats revealed that ZPHA-VRC NPs significantly improved the pharmacokinetic profile of voriconazole, with a 2.6-fold increase in Cmax and a 3-fold increase in bioavailability compared to the free drug. Enhanced distribution to key organs such as the brain and lungs was also observed. In conclusion, the ZPHA-VRC NPs formulation demonstrated superior pharmacokinetic properties and improved bioavailability compared to free voriconazole, indicating its potential as a more effective oral delivery system for this antifungal agent. The enhanced drug concentrations in critical sites like the lungs and brain suggest that nanoparticle-based delivery could offer more effective treatment options for severe fungal infections, such as systemic candidiasis, invasive pulmonary aspergillosis, and cryptococcal meningitis. These findings highlight the promise of ZPHA-VRC NPs as a potent alternative to conventional formulations, with the potential to significantly improve therapeutic outcomes.
